# Management of diffuse large B‐cell lymphoma with cardiac and extra‐cardiac locations

**DOI:** 10.1002/jha2.467

**Published:** 2022-05-09

**Authors:** Pauline Rottier, Pierre‐Marie Morice, Mathieu Bellal

**Affiliations:** ^1^ Hematology Institute University Hospital of Caen Caen France; ^2^ Normandie Univ UNICAEN INSERM U1086 ANTICIPE (Interdisciplinary Research Unit for Cancers Prevention and Treatment) Caen France; ^3^ Department of Intensive Care Unit University Hospital of Caen Caen France; ^4^ Normandie Univ UNICAEN INSERM UMRS U1237 PhIND Caen France

1

A 71‐year‐old man presented with 6 kg weight loss since 4 months and asthenia. He also had mild cough for a few weeks, sudden stabbing thoracic pain recurring at rest without radiation, and no dyspnea. The patient had a history of chronic weaned alcoholism, active smoking, hypertension on triple therapy, and professional exposure to paint. The last transthoracic echocardiography (TTE) performed 6 months before his admission showed an isolated septal hypertrophy at 13 mm without supplementary cardiac abnormalities. At admission, hemoglobin was 138 g/L, platelet and leukocyte counts were 369 × 10^9^/L and 10.1 × 10^9^/L, respectively, C‐reactive protein was 51 mg/L, lactate dehydrogenase (LDH) was 8.57 μkat/L, and troponin I was 86.6 μg/L. Neither clinical nor electrocardiogram additional abnormalities were identified. TTE exhibited a significant necrotic intracardiac mass. Computed tomography (CT) highlighted an 80 × 80 mm infiltrating heart mass centered on the right atrioventricular septum with extension to the tricuspid ring (Figure 1, left image), a mediastino‐hilar lymphadenopathy, a 15 mm right posterobasal pulmonary nodule, and a thin layer of right pleural effusion. A biopsy of the mediastinal lesion by endoscopic ultrasound revealed large B cells, strongly positive for CD20 antigen as well as CD19, CD5 (low intensity), CD10, CD38, CD79b, and FMC7 marker proteins with a Ki‐67 of 70%. Although biopsy was not large, the pathological diagnosis suggested a diffuse large B‐cell lymphoma. During hospitalization, a repeated CT showed increased necrotic areas in mediastinal mass, layer of right pericardial effusion, right posterobasal pulmonary nodule, bilateral pleural effusions with passive atelectasis precluding another biopsy, and hepatosplenomegaly. After occurrence of second‐degree atrioventricular (AV) block, a pacemaker was implanted in the right ventricular apex. Overall, these clinical, radiological, and histological observations were consistent with diagnosis of diffuse large B‐cell lymphoma (DLBCL). Due to the accelerated progression of disease, patient started a first chemotherapy cycle without definitive histological diagnosis including intravenous cyclophosphamide, vincristine, and prednisone without anthracycline. Following six cycles of R‐CHOP (Rituximab, Cyclophosphamide, Hydroxy‐doxorubicin, Vincristine and Prednisone) combination regimen for treatment of DLBCL with extranodal involvement, CT scan showed highly significant regression of cardiac tumors masses (Figure 1, right image artifacted due to the pacemaker) as well as pleural effusions, lymphadenopathy, pulmonary mass, and hepatosplenomegaly. A complete metabolic response was highlighted by positron emission tomography 8.6 months after the first day of CVP.

To date, both primary and secondary cardiac lymphomas (CL) remain aggressive diseases, reported in less than 1% and 10% of patients with DLBCL, respectively. We acknowledged that additional sampling procedures were precluded by the concurrence of rapid progression of DLBCL in a few weeks associated with the onset of high‐grade AV block. Therefore, it remained difficult to definitively establish the primary origin of CL versus secondary cardiac involvement of lymphoma, although timeline of clinical and radiological events and the histopathological data argue for a primary CL in the first instance. Despite an improvement of cardiac lymphoma diagnosis through multimodal imaging, biopsy techniques, and chemotherapy regimen including rituximab, the prognosis of CL patients remains poor.

## CONFLICT OF INTEREST

The authors declare they have no conflicts of interest.

2

## ETHICS STATEMENT

Written informed consent was obtained from the patient.

3

**FIGURE 1:**
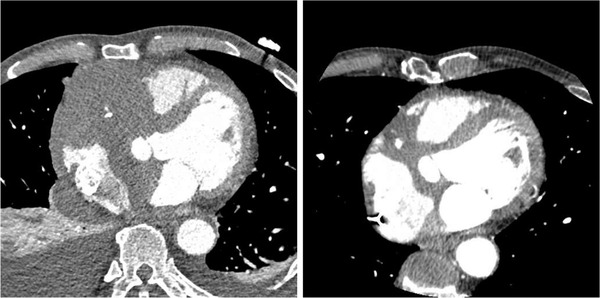
Computed tomography images of the chest, showing extensive lesions in the right atrioventricular septum before (left) and after (right) the first line of chemotherapy, with artifacts due to the pacemaker

## Data Availability

The data that support the findings of this study are available from the corresponding author upon reasonable request.

